# Supplementary figures and images for: Quantitative definition of neurobehavior, vision, hearing and brain volumes in macaques congenitally exposed to Zika virus
Source: PLoS One. 2020 Oct 22;15(10):e0235877. doi: 10.1371/journal.pone.0235877 (PMC7580995; doi:10.1371/journal.pone.0235877)

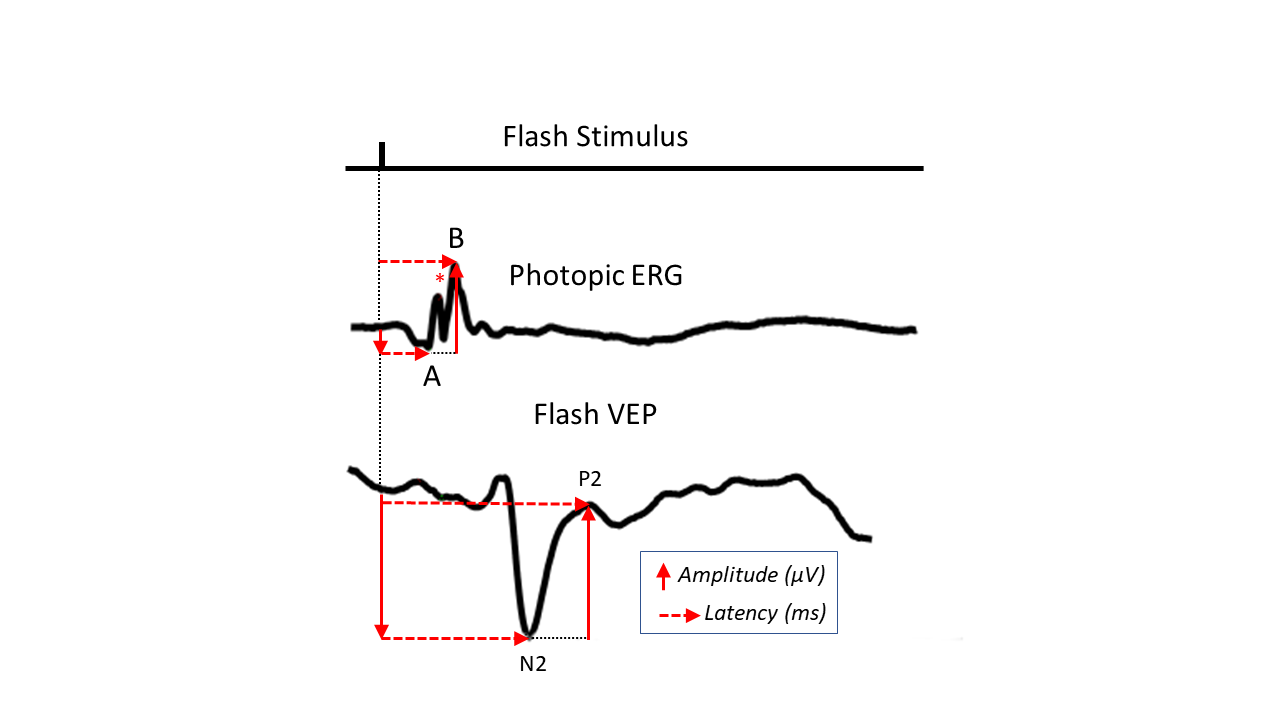

Supplement: S1 Fig — These ERG and VEP waveforms were recorded simultaneously from control infant r18076. Red solid arrows: amplitude of labelled waveform features. ERG A- and VEP N2-wave amplitudes are measured relative to pre-flash voltage; ERG B-and VEP P2-wave amplitude measured from preceding A- and N2-waves, respectively (horizontal dotted line). Red dashed arrows: latency-to-peak, all measured from the onset of the flash stimulus (vertical dotted line). The neonatal rhesus photopic ERG contains a prominent short-latency wave (*) prior to the B-wave that decreases in prominence with age and was not quantified for this study. (TIF) [file pone.0235877.s001.tif]

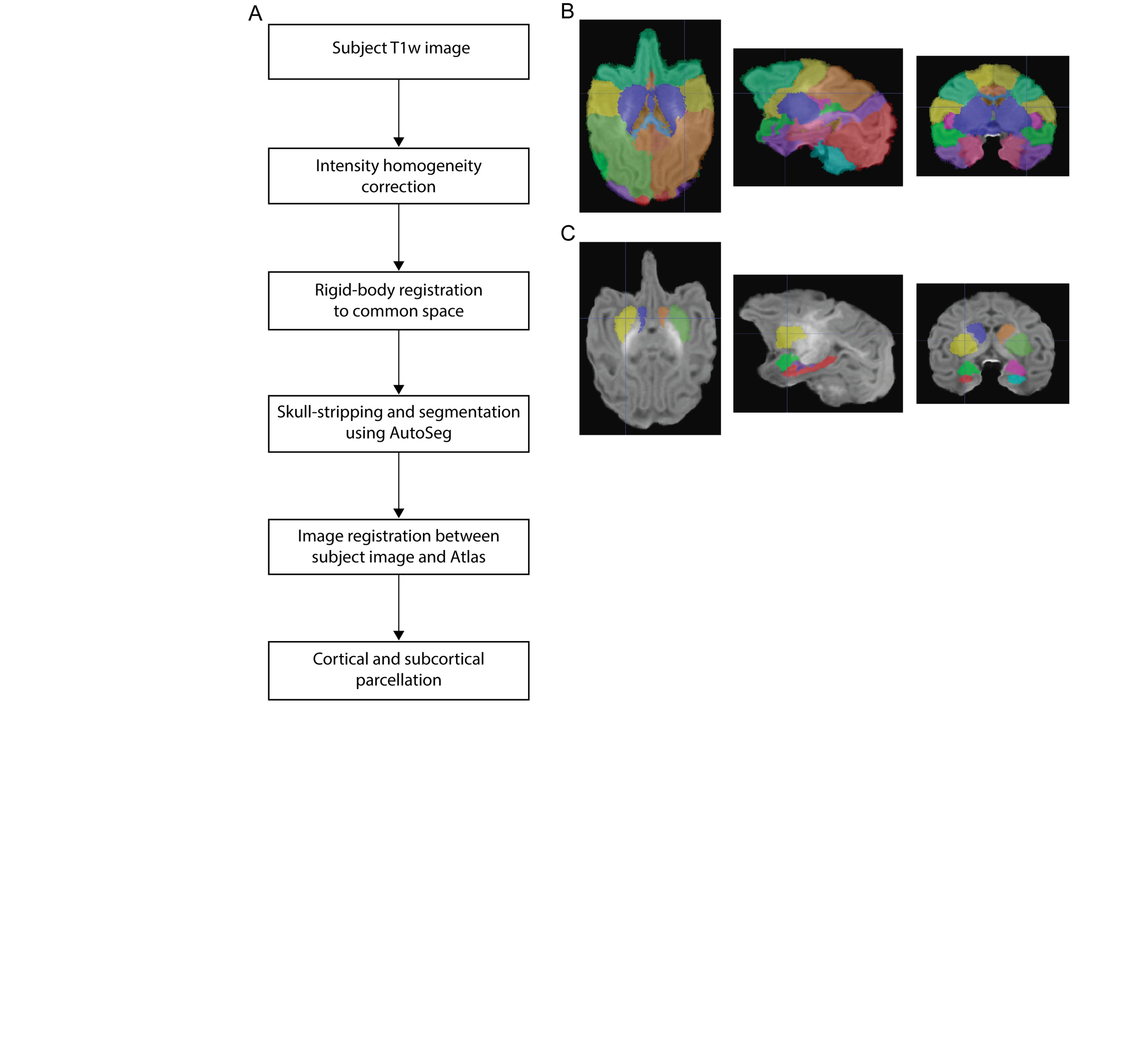

Supplement: S2 Fig — (A) AutoSeg grey matter parcellation with axial (left), sagittal (middle) and coronal (right) views. (B) AutoSeg subcortical structure parcellation with axial (left), sagittal (middle) and coronal (right) views. Parcellations are overlaid on the subject T1w image, registered to the Emory-Cross 2 week infant template. (TIF) [file pone.0235877.s002.tif]

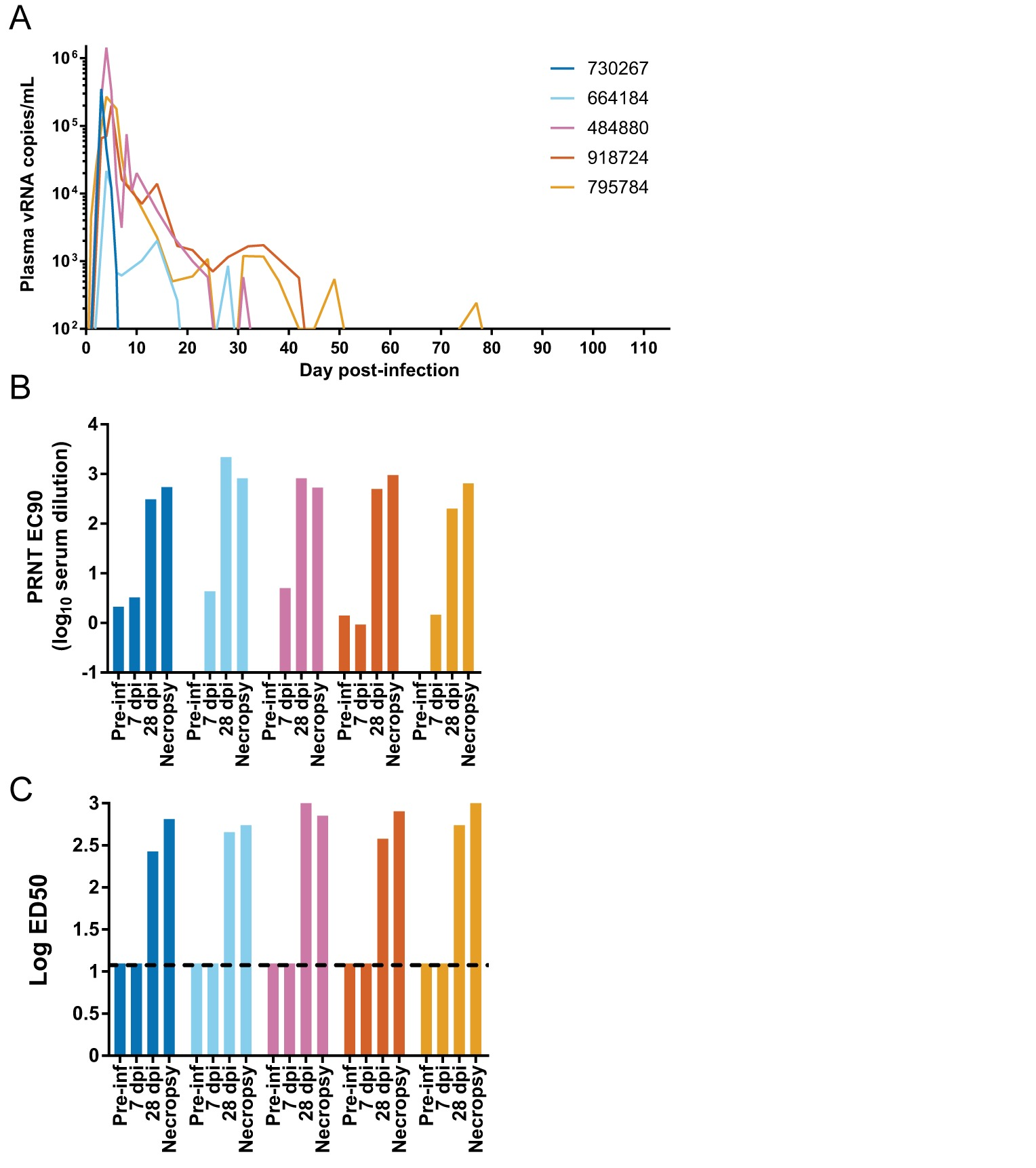

Supplement: S3 Fig — (A) Maternal plasma vRNA loads were measured by qRT-PCR. (B) Estimated EC90 values for serum neutralization of ZIKV assessed prior to infection, 7 dpi, 28 dpi and at maternal necropsy. (C) Estimated EC50 values for serum ZIKV IgG titers obtained prior to infection, 7 dpi, 28 dpi and at maternal necropsy. The dashed line indicates the limit of detection. (TIF) [file pone.0235877.s003.tif]

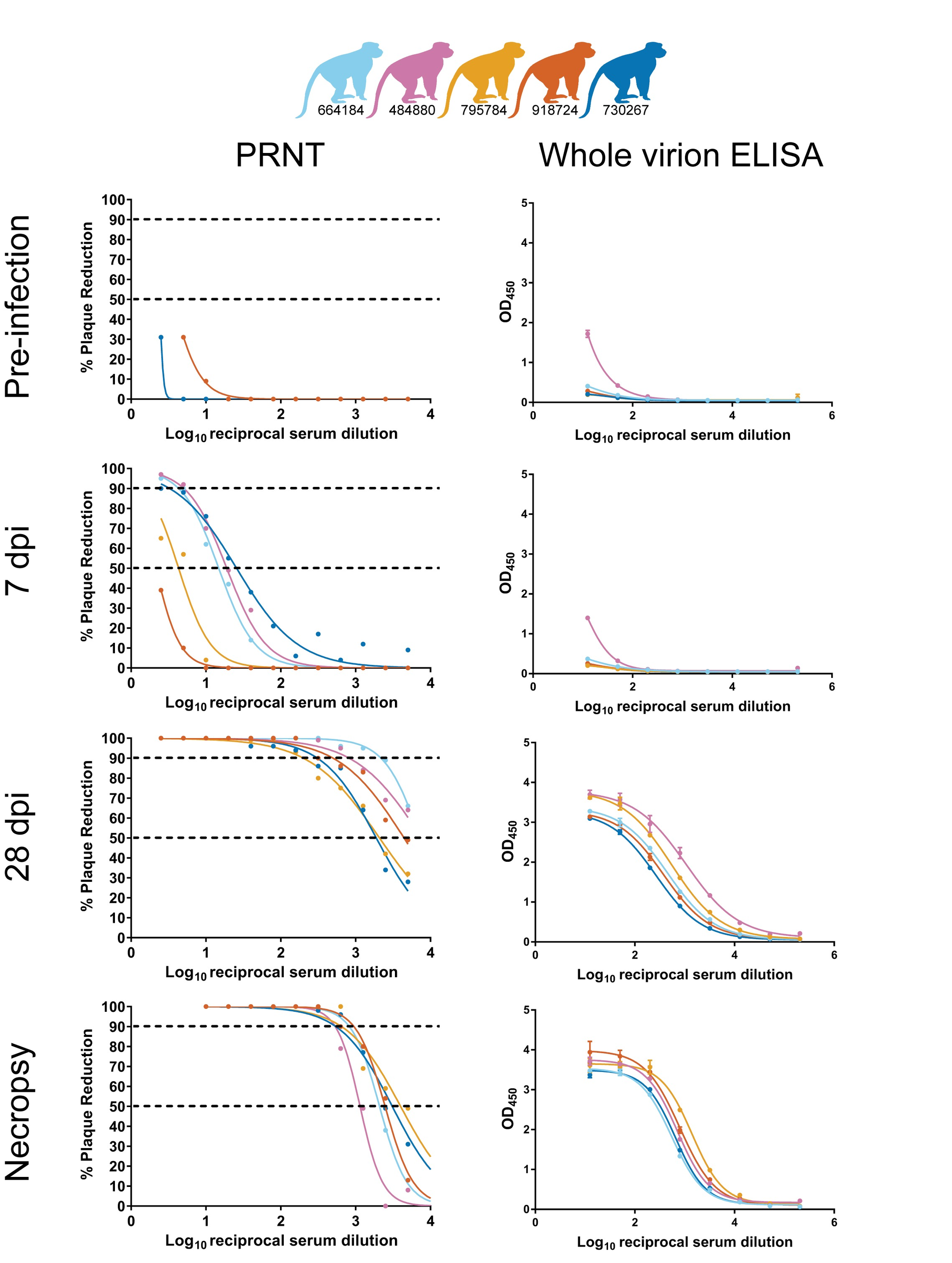

Supplement: S4 Fig — (TIF) [file pone.0235877.s004.tif]

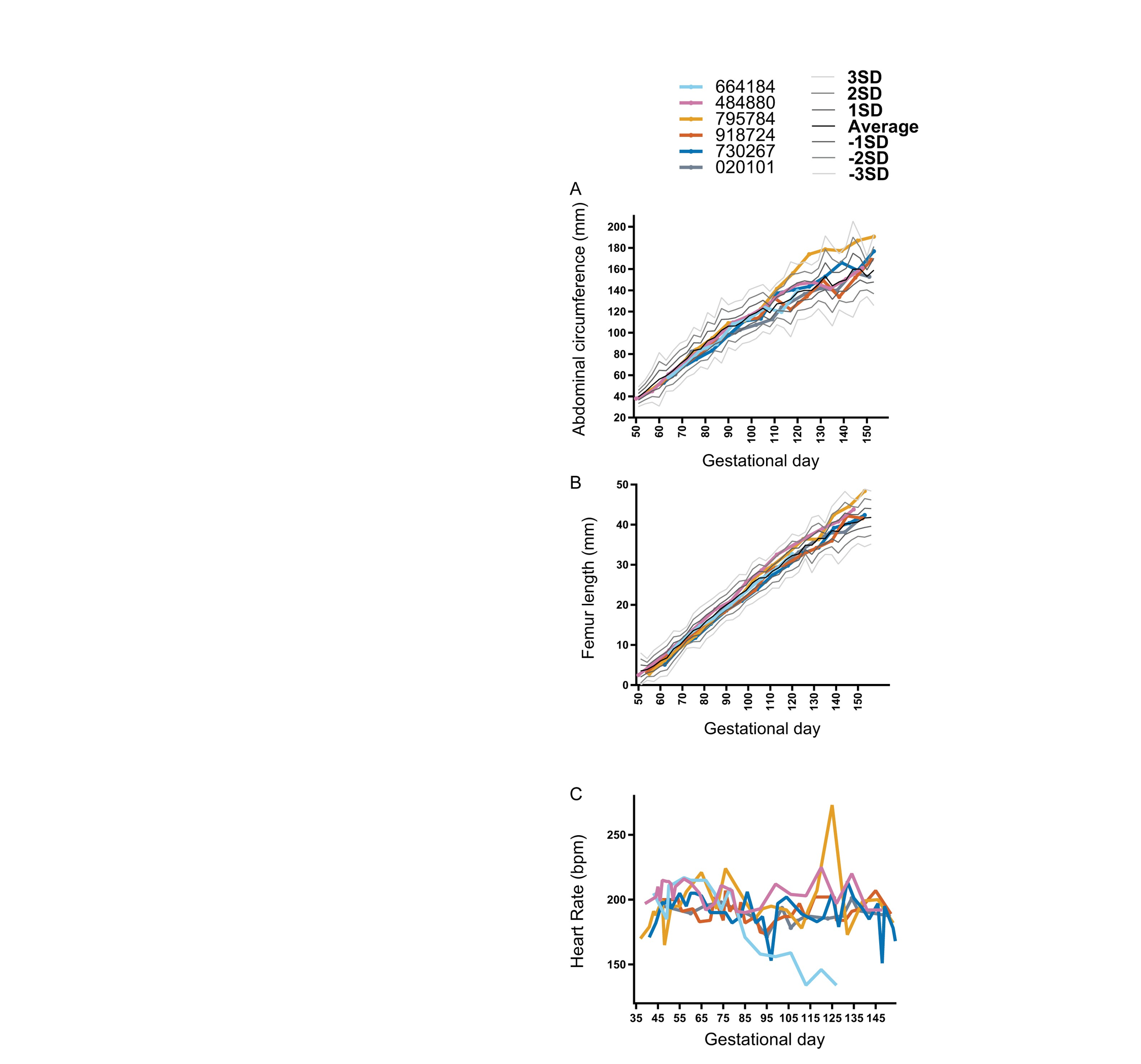

Supplement: S5 Fig — (TIF) [file pone.0235877.s005.tif]

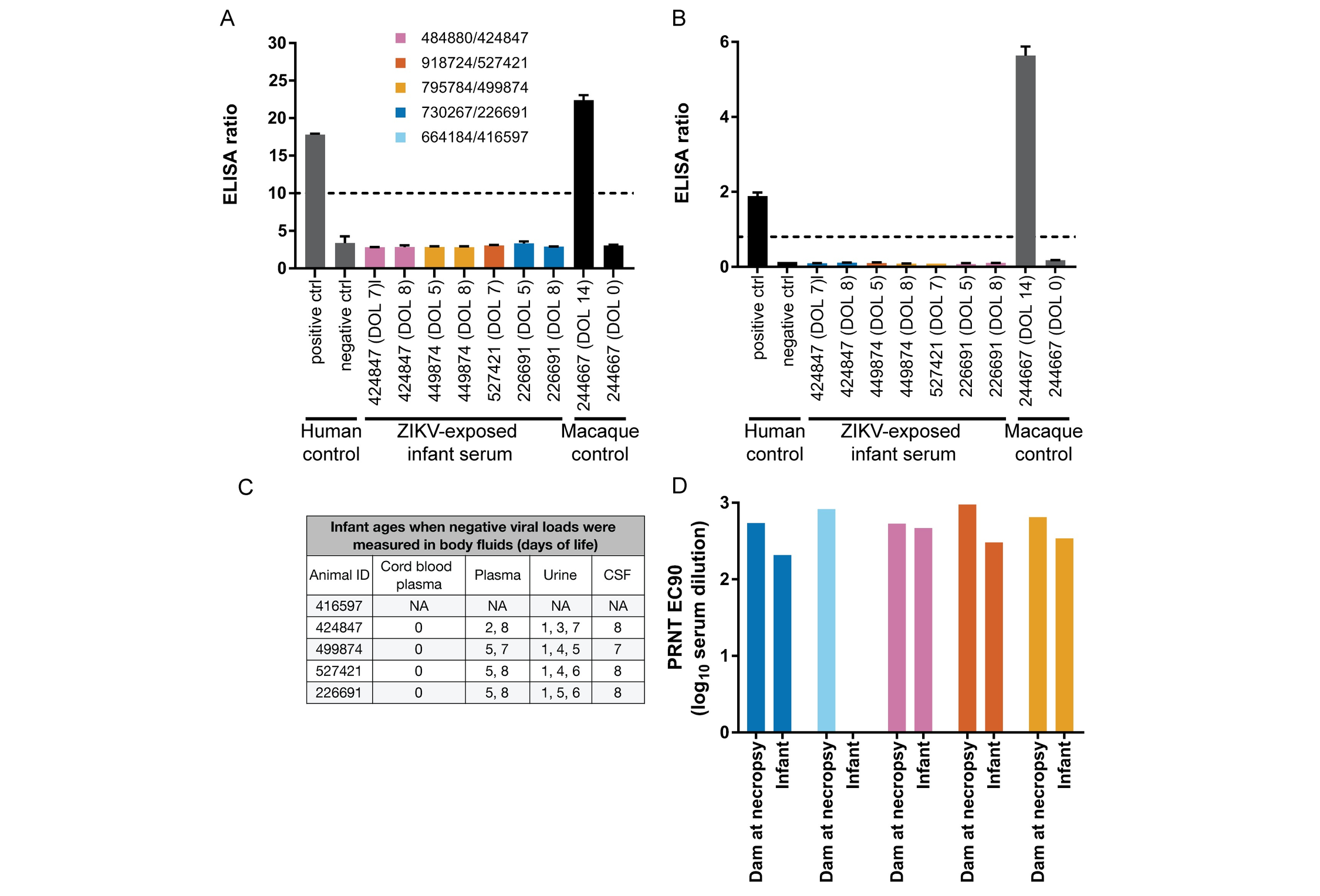

Supplement: S6 Fig — Sera from ZIKV-exposed infants were evaluated for the presence of ZIKV-specific IgM by two commercial assays: Abcam (A) and Euroimmun (B). The manufacturer provided positive and negative human IgM controls are shown in grey. Pre and post-ZIKV infection serum collected from an adult rhesus macaque (244667) was used as a species-specific positive and negative control (shown in black). The dashed line indicates the positive cutoff value for each assay. (C) Infant body fluids (plasma, urine, CSF) were assessed for the presence of ZIKV RNA by qRT-PCR at the days indicated and were negative. NA, not applicable (because no samples were available from the stillborn fetus). (D) PRNTs were performed on infant serum from day of life (DOL) 2–5 from liveborn infants and ED90 values were reported in comparison with maternal PRNT ED90 values from necropsy. (TIF) [file pone.0235877.s006.tif]

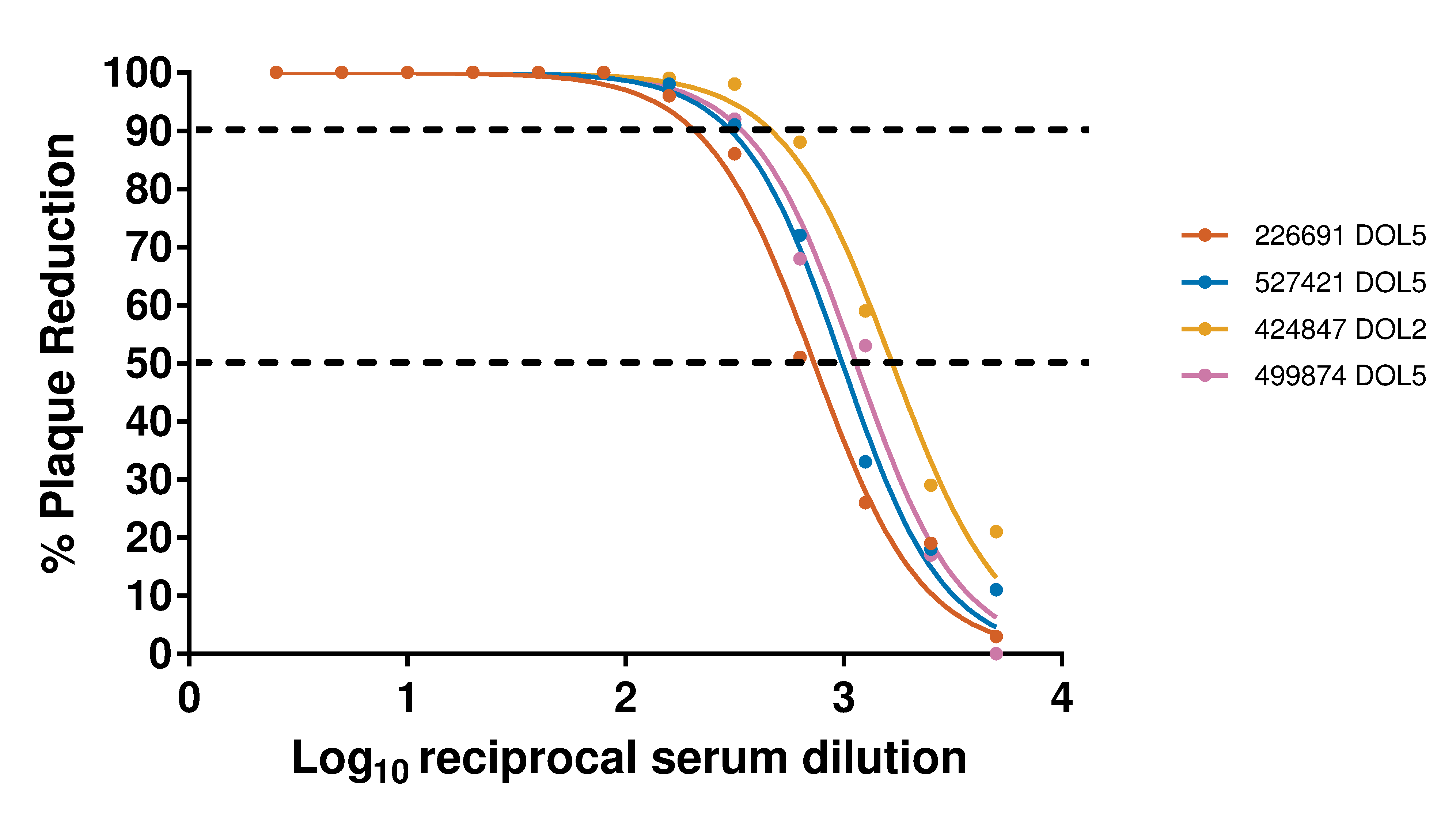

Supplement: S7 Fig — Infant serum was collected at the day of life (DOL) indicated. (TIF) [file pone.0235877.s007.tif]

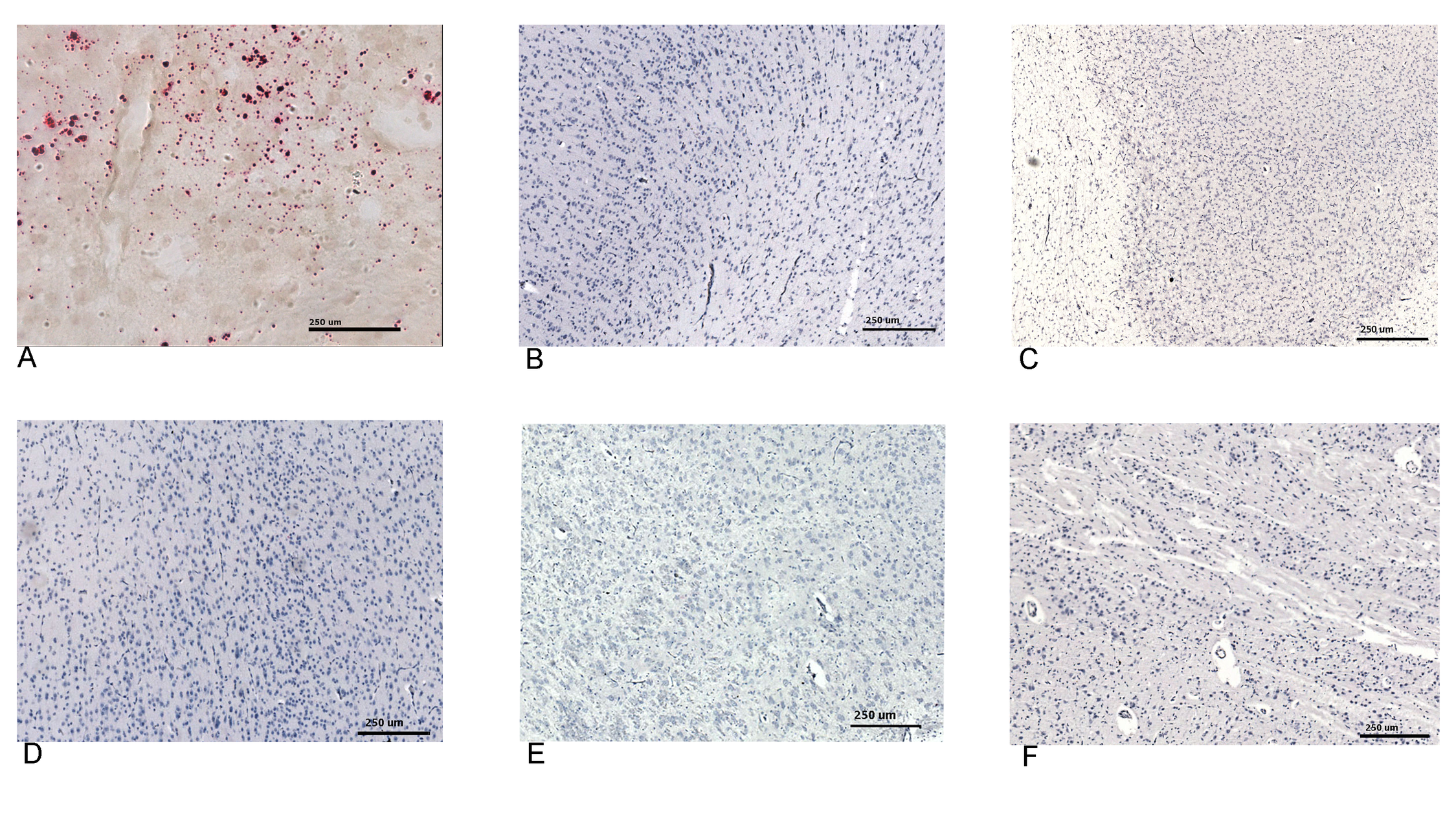

Supplement: S8 Fig — (A) ZIKV genomic RNA (red dots) in a ZIKV-exposed murine lateral geniculate brain section (20x). ZIKV-exposed fetus/infant brain sections exposed to ZIKV RNA probe (10x) (B) 527421 cerebral cortex (rostral to LGN), (C) 499874 cerebral cortex, (D) 226691 cerebral cortex, (E) 424847 cerebrum containing the lateral geniculate nucleus, (F) 416597 cerebellar brain section. (TIF) [file pone.0235877.s008.tif]
