# Supplementary material for: Quantitative definition of neurobehavior, vision, hearing and brain volumes in macaques congenitally exposed to Zika virus
Source: PLoS One. 2020 Oct 22;15(10):e0235877. doi: 10.1371/journal.pone.0235877 (PMC7580995; doi:10.1371/journal.pone.0235877)
Supplement: S1 Appendix — (DOCX) [file pone.0235877.s009.docx]

**S1 Appendix.** Effect size and post-hoc sample size calculations SAS code.

*Read in raw data (csv format);

proc import file="data.csv" dbms=tab out=input;

data input;

set input;

proc sort;

by parameter group;

proc means noprint;

var value;

by parameter group;

output out=calcMean mean(value)=M SD(value)=SD N(value)=N;

run;

data control;

set calcMean;

if group="Control";

M1=M;

SD1=SD;

N1=N;

keep parameter M1 SD1 N1;

data Zika;

set calcMean;

if group="Zika ";

M2=M;

SD2=SD;

N2=N;

keep parameter M2 SD2 N2;

*Calculate Effect Sizes and conduct Post-Hoc Sample Size calculation;

data final;

merge control Zika;

by parameter

pooledSD=sqrt(((N1-1)*SD1**2+(N2-1)*SD2**2)/(N1+N2-1));

ES=abs(M2-M1)/pooledSD;

*Sample Size 80% power at two sided 0.05 significance level;

Ncalc=ceil(2*(((quantile("normal",0.8))

+quantile("normal",1-0.05/2))/ES)**2);

keep parameter ES Ncalc;

*Export estimated effect sizes;

proc export file="dataEffectSize.txt" replace;

run;
