# Supplementary material for: Quantitative definition of neurobehavior, vision, hearing and brain volumes in macaques congenitally exposed to Zika virus
Source: PLoS One. 2020 Oct 22;15(10):e0235877. doi: 10.1371/journal.pone.0235877 (PMC7580995; doi:10.1371/journal.pone.0235877)
Supplement: S3 Table — (DOCX) [file pone.0235877.s012.docx]

S3 Table. Infant procedural sedation medication record.

| Infant ID | Examination | Procedural sedation regimen |
| --- | --- | --- |
| 424847 | Brain MRI | Ketamine |
|  | Eye exam | ketamine, dexmedetomidine |
| 499874 | Brain MRI | ketamine, midazolam, propofol |
|  | Eye exam | midazolam, propofol, midazolam |
| 527421 | Brain MRI | propofol, isoflurane, midazolam, ketamine |
|  | Eye exam | propofol |
| 226691 | Brain MRI | isoflurane, midazolam, ketamine |
|  | Eye and hearing exam | isoflurane, ketamine, propofol, dexmedetomidine |
| 020501 | Brain MRI and hearing exam | dexmedetomidine, propofol |
|  | Eye exam | propofol, isoflurane, midazolam, ketamine |

Magnetic resonance imaging (MRI).
